# Case Report: Multi-Modality Imaging of a Right Atrial Pseudoaneurysm in a Patient With Breast Cancer

**DOI:** 10.3389/fcvm.2020.623580

**Published:** 2021-01-15

**Authors:** Ying Zhong, Chun-yan Ma, Xu Dai, Guan Wang

**Affiliations:** ^1^Department of Radiology, The First Affiliated Hospital of China Medical University, Shenyang, China; ^2^Department of Echocardiography, The First Affiliated Hospital of China Medical University, Shenyang, China

**Keywords:** right atrial pseudoaneurysm, cardiac magnetic resonance imaging (CMR), computed tomography (CT), echocardiography, case report

## Abstract

Cardiac pseudoaneurysms occur when a blood vessel wall is injured and the leaking blood is collected in the surrounding tissue. They are very rare events and have a high risk of rupture and poor prognosis. We report a case of right atrial pseudoaneurysm in a 54-year-old female patient diagnosed with breast cancer and lung metastasis. The patient underwent five intrapericardial infusions of cisplatin and nine cycles of systemic chemotherapy. Non-contrast-enhanced computed tomography (CT) was performed at follow-up evaluation during the chemotherapeutic process as this patient was contraindicated to iodine. CT without contrast and ultrasonography showed a crescent-shaped lesion near the right atrium but its nature could not be determined. Cardiac magnetic resonance (CMR) imaging with gadolinium contrast provided important information as an alternative enhanced imaging modality. By combining CT, ultrasonography and CMR images with the medical history of the patient, we inferred that the lesion was a pseudoaneurysm in the right atrium. This condition was related to the erosion of metastasized tumor cells or the accumulated cardiac toxicity of multiple cycles of chemotherapy or pericardiocentesis. This single case report suggests that cardiac rupture should be considered as a potential complication in patients with suspected pericardial metastasis. CMR imaging is an excellent tool for the detection of right atrial rupture.

## Case Description

A 54-year-old female patient diagnosed with breast cancer and lung metastasis underwent five intrapericardial cisplatin infusions and nine cycles of systemic chemotherapy. After her first admission, lung enhanced CT and echocardiography showed a large amount of pericardial effusion (PE). The patient developed chest tightness and shortness of breath. Pericardiocentesis showed cancer cells. A large amount of bloody PE recurred after being drained by a pericardial catheter. After comprehensive evaluation intrapericardial chemotherapy was prescribed for the patient.

Through pericardial catheterization, cisplatin was injected into the pericardium. The PE gradually reduced and did not recur. The patient's symptoms also improved significantly. The drainage tube was removed and the intrapericardial chemotherapy was stopped. Systemic chemotherapy was then performed. According to the status of the patient and evaluation of the size of cancer lesions, the chemotherapy program was adjusted. The main drugs used in the process of systemic chemotherapy include albumin, paclitaxel, Xeloda, enantone, anastrozole, vinorelbine, cisplatin, exemesolen, and iverolimus. During chemotherapy, the patient did not feel any obvious discomfort.

**Figure 1:**
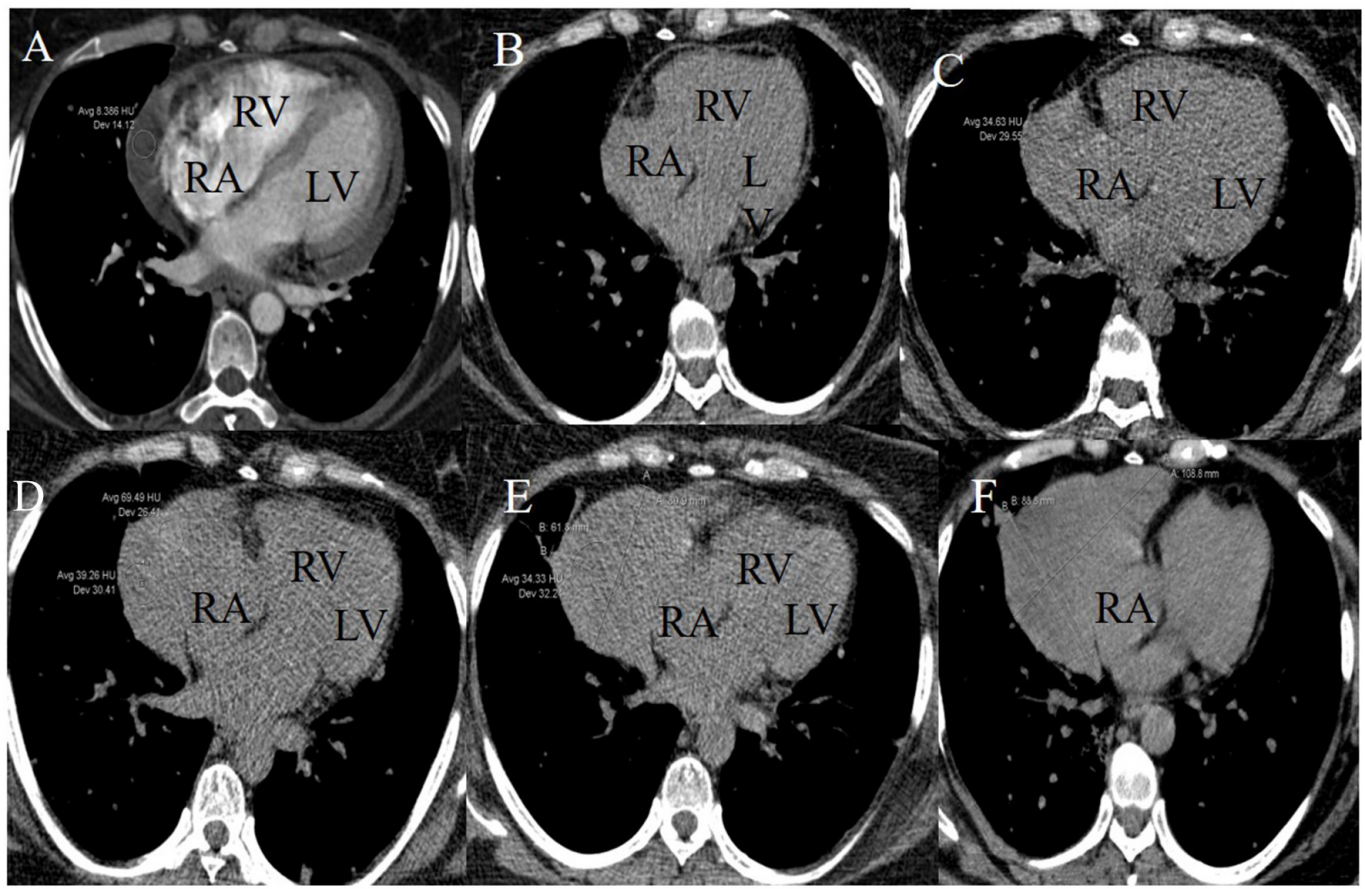
Longitudinal right atrial pseudoaneurysm development on lung CT images. **(A)** A baseline CT image (enhanced) scanned before initiating the intrapericardial chemotherapy revealed PE and intact RA free wall. **(B)** A non-contrast-enhanced CT image scanned after five intrapericardial and three systemic cycles of chemotherapy demonstrated decreased PE and intact RA free wall. **(C–F)** Non-contrast-enhanced CT images after the fifth, sixth, seventh, and ninth systemic cycles of chemotherapy showed the growth of a crescent-shaped soft tissue lesion near the RA. The lesion density after the fifth systemic chemotherapy cycle was mixed.

Follow-up was performed by non-contrast enhanced computed tomography (CT) during the chemotherapeutic process. CT images indicated the presence of a lesion near the right atrium (RA) at the fourth cycle of systemic chemotherapy. The lesion progressively increased in size at subsequent follow-up CT scans. Baseline CT images (enhanced) were compared to scans before intrapericardial perfusion chemotherapy and showed that PE was significant near the RA with a CT value of about eight Hounsfield units (Figure 1A). Non-contrast-enhanced CT images obtained after five cycles of intrapericardial chemotherapy and three cycles of systemic chemotherapy demonstrated decreased PE (Figure 1B). After the fifth, sixth, and seventh cycles of systemic chemotherapy, the non-contrast-enhanced CT images showed the growth of a crescent-shaped lesion with soft tissue density near the RA (Figures 1C–E). The lesion density after the fifth cycle of systemic chemotherapy was mixed (Figure 1D). The boundary between the lesion and the free wall of the RA was unclear and was not typical PE and could potentially have a metastatic tumor.

**Figure 2:**
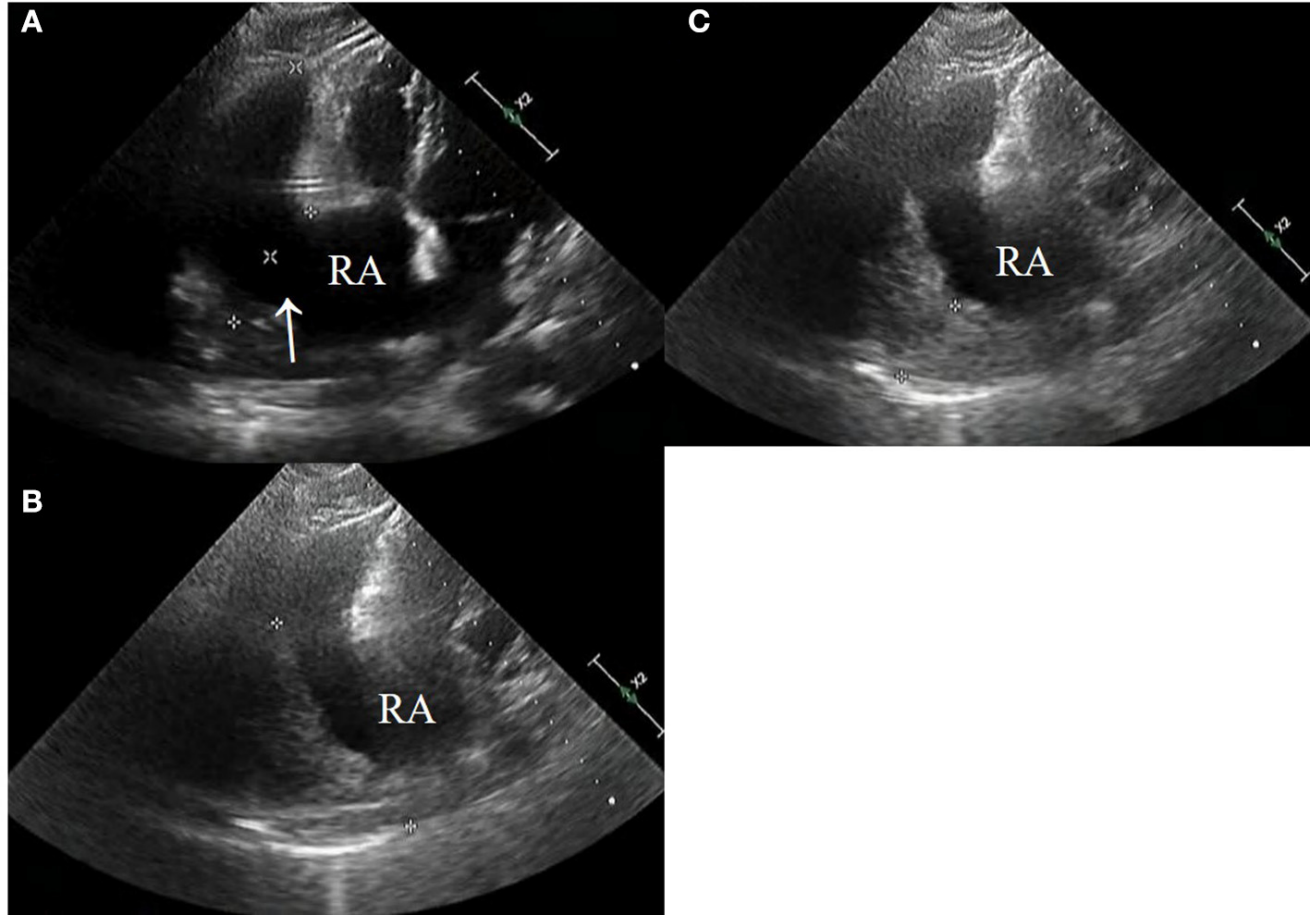
**(A–C)** Echocardiography after the ninth systemic chemotherapy cycle revealed a large rupture in the RA (arrow) that bulged outwards to form a large cyst having a size of ~49 × 71 mm (*). A low echogenic region was attached to the capsule with a range of 98 mm and a thickness of 38 mm (*).

CT images showed further enlargement of the lesion adjacent to the RA. The patient was in a good condition and refused further examinations. After the ninth cycle of systemic chemotherapy, the non-contrast-enhanced CT images showed that the lesion was larger at a size of around 109 × 89 mm (Figure 1F). Transthoracic 3-D echocardiography after the ninth cycle of systemic chemotherapy detected an anechoic area measuring 49 × 71 mm attached to the lateral side of the RA (Figure 2A). There was a weak echo in the area with a range of 98 mm and a thickness of ~36 mm (Figures 2B,C). The pericardium at the apex of both ventricles was also thickened.

**Figure 3:**
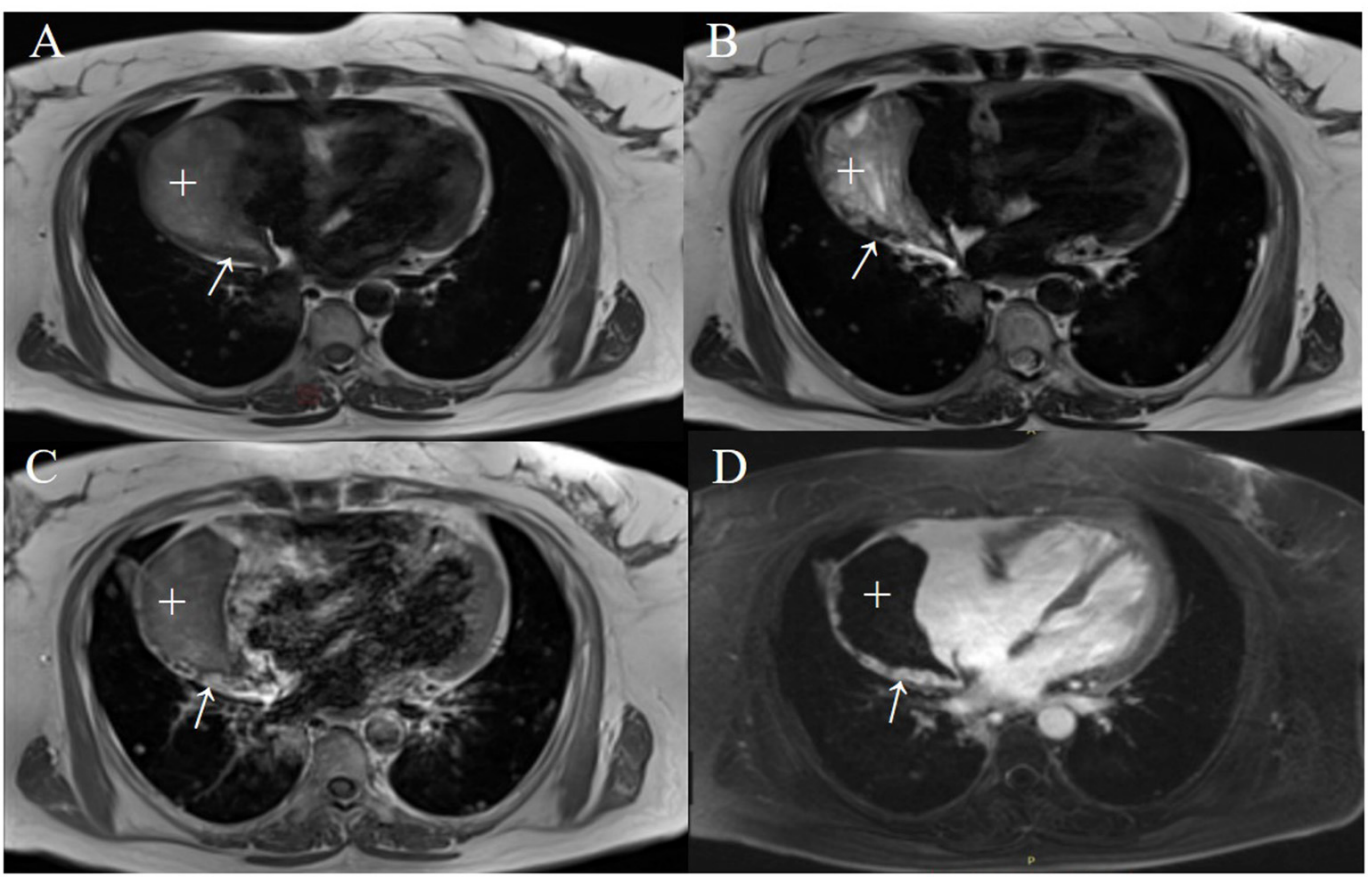
The right atrial pseudoaneurysm with a mural thrombus on CMR images acquired after the ninth systematic chemotherapy cycle. The T1-weighted (T1WI) **(A)** and T2-weighted (T2WI) **(B)** images in a 4-chamber view revealed a crescent-shaped heterogeneous signal of 87 × 43 mm in size (+) and a striped isogenic signal (thickened pericardium) of 3–8 mm in width (arrows). An early enhanced T1WI image **(C)** and late gadolinium-enhanced imaging **(D)** illustrate diffuse enhancement within a striped lesion.

Based on the CT and echocardiography images and to further clarify the characteristics of the lesion due to clinical concern of a possible RA rupture, cardiac magnetic resonance (CMR) imaging was conducted to obtain a definitive evaluation and tissue characterization. The CMR images (as shown in Figure 3) revealed an extra cavity adjacent and connected to the RA with an extensive mass adherent to the right wall of the cavity. The flow from the RA cavity through the crevasse into the pseudoaneurysm cavity and the flow disturbances around the residual RA ruptured wall were directly presented on the Cine images (Supplementary Videos 1–4). These findings raised the concern of a possible rupture of the RA free wall with subsequent pseudoaneurysm.

The lateral pericardial wall of the cystic cavity was thickened to around 3–8 mm. It could be viewed with iso-T1 and iso-T2 signals and diffuse enhancement was observed in the early and delayed phases after administration of a gadolinium contrast agent. Based on these findings the diagnosis may be a pericardial metastasis of the tumor. The Cine images also implied that the right ventricle basement was compressed and its free wall was oscillating (Supplementary Videos 1–4).

From the observations, the patient underwent contrast-enhanced echocardiography examination which also indicated that the right atrial wall was ruptured forming a pseudoaneurysm. The presentation of contrast-enhanced echocardiography was basically consistent with CMR but did not showed the enhancement of the lateral pericardial wall of the pseudoaneurysm. The dynamic images of the contrast-enhanced echocardiography (Supplementary Video 5) are shown in the Online Appendix.

## Discussion

Cardiac pseudoaneurysms typically originate in the left ventricle. There have been a few reports of right atrial pseudoaneurysm appearing after cardiac surgery (1) or blunt trauma (2). Cardiac rupture generally leads to massive hemorrhage and high mortality and so this condition is not often diagnosed antemortem. In this case, the patient presented with a chronic injury process. Because the pressure in the RA was low and the atrial rupture was initially small, the blood may have leaked slowly, thereby avoiding a fatal pericardial tamponade due to massive amounts of blood leaking into the pericardial sac within a short time.

After this, the rupture was relatively large due to the growth of the pseudoaneurysm. The blood may have been able to communicate between the pseudoaneurysm and the residual cavity of the RA rather than a one-way throttle that can easily cause cardiac tamponade. In addition, the wall-attached thrombus passively stabilized the aneurysm thereby preventing sudden death caused by the pseudoaneurysm rupture. The progressive enlargement of the lesion viewed in repeated follow-up CT images supports this hypothesis.

Our observations indicated that not all pseudoaneurysms need immediate surgery. In cases of chronic cardiac pseudoaneurysms as observed in this patient, conservative treatment may be a better choice. This patient experienced a severe adverse reaction after iodine contrast administration during the baseline lung CT scans. Only non-contrast-enhanced lung CT scans were acquired during the subsequent follow-up evaluations.

The patient refused further invasive treatment as the breast cancer had progressed to end stage disease. This decision made antemortem identification of the cause of atrial rupture difficult. However, considering the patient's medical history and treatment process, the following reasons were suspected: (1) considering the pericardial thickening, diffuse enhancement, and cancer cells in the bloody PE, tumor cells may have metastasized into the pericardium and further eroded the adjacent RA myocardium. The slow-leaking hemorrhage was capsulated by the metastasizing pericardium and eventually resulted in a pseudoaneurysm. (2) Secondly, multiple catheterizations and pericardiocentesis could potentially lead to accidental atrial wall perforation. Several studies have shown that the incidence of complications of pericardiocentesis is 3% and the incidence of iatrogenic ventricular rupture is 1% (3). Pericardiocentesis mainly causes right ventricular perforation (4). To some extent, this highlighted that multiple catheterization and pericardiocentesis may lead to accidental atrial wall perforation in this patient, which belongs to iatrogenic complications. (3) Finally, the cardiotoxic side effects might induce injury of the myocardium during systemic chemotherapy. Previous studies have demonstrated that heart injury caused by chemotherapeutic drugs (5) and cardiac toxicity is mostly related to dose accumulation (6). In this case, paclitaxel, cisplatin, and other chemotherapeutic drugs used during cycled chemotherapy exhibited strong myocardial toxicity. Although cardiovascular events are not common complications, they mainly presented as myocardial infarction (7, 8) and were likely to result in cardiac rupture (9). We hypothesized that the above three mechanisms are the most probable causes of right atrial pseudoaneurysm formation in this patient.

## Diagnosis

Surgical evaluation is the gold standard for the diagnosis of pseudoaneurysms. However, in the absence of pathology, it is necessary to assess the lesions using a variety of imaging methods to differentiate pseudoaneurysms from true aneurysms. Cardiac pseudoaneurysms are usually a cystic cavity formed in the pericardium or proliferative fibrous tissue after local rupture of the heart which is often accompanied by mural thrombosis. However, true aneurysms are usually characterized by weakening of the local myocardial structure that results in bulging cysts. The ratio of the long diameter of the breach to the maximum diameter of the pseudoaneurysm is usually <50% whilst true aneurysms have a broad base (10). In this case, CT, ultrasound and CMR images of the lesion showed that the rupture of the RA was connected with the lesion and mural thrombus could be seen inside. The base of the lesion was narrow and the ratio to the widest part of the lesion was <50%. Combined with the patient's history and the imaging findings, a diagnosis of pseudoaneurysm of the RA was given.

Imaging diagnosis of cardiac pseudoaneurysm is complex particularly in the RA. Echocardiography is widely used due to its convenience and wide availability. However, the geometry of the right side of the heart and its position in the chest make it challenging to assess structure and function using echocardiographic evaluation (11) unless the lesion is extensive and obvious. CMR and cardiac CT can be used to observe any plane of the heart. Enhanced cardiac magnetic resonance imaging (MRI) is highly reproducible and largely operator-independent, allowing precise differentiation and tissue characterization. Some patients are hypersensitive to the contrast agents or may be contraindicated to the CMR scan. Hence, multi-modality imaging is more appropriate and provides benefit to this group of patients.

## Treatment

Pseudoaneurysms have a high risk of rupture and can easily cause cardiac tamponade that can endanger the lives of patients and require immediate surgical repair. However, this patient did not have any symptoms of pericardial tamponade. Therefore, our observations indicated that not all pseudoaneurysms need immediate surgery immediately especially for a chronic cardiac pseudoaneurysm in a terminal period cancer patient. In this case conservative treatment may be a better option. In a study from Yeo et al. (12) that reported long-term follow-up in 52 cases of cardiac pseudoaneurysms, some patients did not undergo surgery and the cardiac pseudoaneurysms did not rupture, which agreed with this case study. In our case, the patient continued to have chemotherapy and was followed up with CMR and echocardiography to observe the development of a right atrial pseudoaneurysm.

## Limitations

The patient refused surgery and we could not determine the real cause of the formation of the atrial pseudoaneurysm.

## Conclusions

Cardiac pseudoaneurysms should be diagnosed by combining imaging and medical history when pathology is not available. Not all cardiac pseudoaneurysms require immediate surgery, especially chronic cardiac pseudoaneurysms. This single case report suggests that common myocardial infarction and trauma are not the only possible causes of cardiac rupture. CMR is an excellent tool to diagnose and identify the causes of RA free wall rupture particularly in patients contraindicated to iodine contrast medium.

## Data Availability Statement

The original contributions generated for the study are included in the article/Supplementary Material, further inquiries can be directed to the corresponding authors.

## Ethics Statement

The studies involving human participants were reviewed and approved by Department of Ethics Committee for Medical Science Research, the First Affiliated Hospital of China Medical University, Shenyang, China. The patients/participants provided their written informed consent to participate in this study.

## Author Contributions

All authors listed have made a substantial, direct and intellectual contribution to the work, and approved it for publication.

## Conflict of Interest

The authors declare that the research was conducted in the absence of any commercial or financial relationships that could be construed as a potential conflict of interest.

## Supplementary Material

Supplementary Videos 1–4 The flow from the RA cavity through the crevasse into the pseudoaneurysm cavity and the flow disturbances around the residual RA ruptured wall were directly presented on the Cine MR images. The Cine images also showed that the right ventricle basement was compressed and its free wall was oscillating.

Supplementary Video 5 Dynamic images of contrast echocardiography indicated that a large anechoic area on the lateral side of the RA where there was a weak echo (the mural thrombus) with an attachment thickness of 40–42 mm. After injection of the contrast agent, the RA and the anechoic area were simultaneously filled with the contrast agent, the mural thrombus was not enhanced. The myocardial contrast mode showed a contrast-enhanced agent filling in the upper part of the mural thrombus in the anechoic area and no obvious contrast-enhanced agent being found in the lower.
